# Endocannabinod Signal Dysregulation in Autism Spectrum Disorders: A Correlation Link between Inflammatory State and Neuro-Immune Alterations

**DOI:** 10.3390/ijms18071425

**Published:** 2017-07-03

**Authors:** Anna Lisa Brigida, Stephen Schultz, Mariana Cascone, Nicola Antonucci, Dario Siniscalco

**Affiliations:** 1 Department of Experimental Medicine, University of Campania, 80138 Naples, Italy; brigida.annalisa@gmail.com; 2 Department of Cellular and Integrative Physiology, School of Medicine, University of Texas Health Science Center San Antonio, San Antonio, TX 78229, USA; stevendri0629@gmail.com; 3 Cascone Health and Nutrition Centre, 80050 S. Maria La Carità, Italy; mariana_1984@live.it; 4 Biomedical Centre for Autism Research and Treatment, 70126 Bari, Italy; info@antonucci.eu

**Keywords:** endocannabinoid system, neuro-immune system, monocyte, autism

## Abstract

Several studies highlight a key involvement of endocannabinoid (EC) system in autism pathophysiology. The EC system is a complex network of lipid signaling pathways comprised of arachidonic acid-derived compounds (anandamide, AEA) and 2-arachidonoyl glycerol (2-AG), their G-protein-coupled receptors (cannabinoid receptors CB1 and CB2) and the associated enzymes. In addition to autism, the EC system is also involved in several other psychiatric disorders (i.e., anxiety, major depression, bipolar disorder and schizophrenia). This system is a key regulator of metabolic and cellular pathways involved in autism, such as food intake, energy metabolism and immune system control. Early studies in autism animal models have demonstrated alterations in the brain’s EC system. Autism is also characterized by immune system dysregulation. This alteration includes differential monocyte and macrophage responses, and abnormal cytokine and T cell levels. EC system dysfunction in a monocyte and macrophagic cellular model of autism has been demonstrated by showing that the mRNA and protein for CB2 receptor and EC enzymes were significantly dysregulated, further indicating the involvement of the EC system in autism-associated immunological disruptions. Taken together, these new findings offer a novel perspective in autism research and indicate that the EC system could represent a novel target option for autism pharmacotherapy.

## 1. Introduction: Autism

According to US National Institute of Mental Health, autism spectrum disorder (ASD) is the name for a group of developmental disorders [1] and is characterized by the diagnostic and treatment manual for mental disorders, fifth edition (DSM-5), as possessing persistent deficits in social communication and interaction, restricted, repetitive patterns of behavior, interests, or activities [2,3]. ASD includes a wide range (the so called spectrum) of symptoms, skills, and levels of disability. However, DSM-5 does not include subcategories of a larger disorder, but the range of characteristics and severity within one category are described [1]. Symptoms of ASD begin in early childhood, and produce clinically significant developmental impairment [2]. Some cases of ASD children display genetic or chromosomal abnormalities as seen in Fragile X syndrome or Down syndrome; however, most cases of ASD have an unknown etiology [1]. Based on most recent prevalence data, worldwide population prevalence is about 1% [4]. Intellectual disability is present in about 45% of individuals with autism and 32% have regression [4].

## 2. Autism and Inflammatory State

The inflammatory state has been shown to be associated with ASD in many studies which show abnormalities in immune system components [5]. A recent study showed that interleukin 8 was significantly increased in blood of children with ASD [6]. Further, immune system disruption [7,8] and immune system dysfunction have been implicated in ASD [9,10,11,12,13,14]. These abnormalities include differential monocyte responses, abnormal T helper cytokine levels, decreased T cell mitogen response, decreased numbers of lymphocytes, and abnormal serum immunoglobulin levels. Arima et al. have shown that regional neuronal activation provides a mechanism by which autoreactive T cells may cross the blood brain barrier [15]. Neutrophils have been shown to mediate disruption of the blood-spinal cord barrier in some neuroinflammatory diseases [16], which may have implications for autism. ASD children display differential monocyte responses to toll-like receptor ligands [17].

Other studies have shown that children with autism exhibit immune system abnormalities, in particular for antibodies against brain and central nervous system proteins, as well as against maternal proteins [18,19,20,21,22,23,24,25], and increased plasma pro-inflammatory cytokine levels [26,27]. The relationship of serum anti-neuronal antibodies and increased autism severity has been demonstrated [28]. Aktas et al. have demonstrated neuronal damage in autoimmune neural inflammation which is mediated by the death ligand TRAIL [29]. Garay et al. have proposed novel roles for immune system molecules in neural development that may have implications for autism [30]. They proposed that major histocompatibility complex I (MHCI) and its receptors, complement, and cytokines influence the function and development of brain synapses and influence the development of ASD. Over-expression and activation of several caspases was found in autistic peripheral blood mononuclear cells [31]. Among them, the mRNA levels of pro-inflammatory caspase-1, -4 and -5 and protein levels of caspase-7 and -12 were significantly increased, along with over-activation of caspase-3.

## 3. Endocannabinoid System

**Figure 1:**
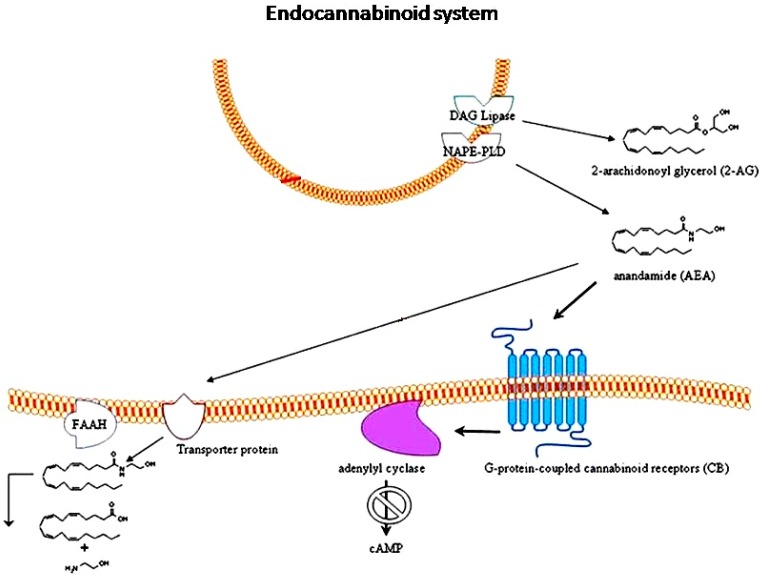
Endocannabinoids, such as *N*-arachidonoylethanolamine (anandamide, AEA) and 2-arachidonoyl glycerol (2-AG), are synthesized and released upon demand in a receptor-dependent way, through the AEA biosynthetic enzyme *N*-acylphosphatidylethanolamine-hydrolyzing phospholipase D (NAPE-PLD) and the diacylglycerol (DAG) lipase enzyme, respectively. They exert their effects through the G-protein-coupled cannabinoid receptors CB1 and CB2, which, in turn, are negatively coupled to adenylyl cyclase enzyme. After the specific binding with their receptors, endocannabinoids are transported into cells by a specific uptake system and degraded by the enzymes fatty acid amide hydrolase (FAAH). Adapted from [47], with permission of Springer.

The endocannabinoid system (EC) is comprised of arachidonic acid derived compounds, their receptors and the associated enzymes (Figure 1) [32]. The EC system represents an intricate network of lipid signaling pathways. The naturally occurring EC “building blocks” are *N*-arachidonoylethanolamine (anandamide, AEA) and 2-arachidonoyl glycerol (2-AG), that exert their effects through the G-protein-coupled cannabinoid receptor (GPCR) type 1 (CB1) and type 2 (CB2), which, in turn, are negatively coupled to the adenylate cyclase enzyme [33]. AEA (whose name derives from “ananda” that in Sanskrit means “joy” [34]) and 2-AG are part of the molecular group of *N*-acylethanolamines (NAEs) and monoacylglycerol (MAG) glycerophospholipids classes [35], and were the first described endogenous ligands of CB receptors [36,37]. The two CB1 and CB2 belong to the class-A GPCR subfamily receptors [38]. They are heptahelical transmembrane receptors in that the N-terminal domain is localized outside the membrane and contains the ligand bound site, whereas the C-terminal domain is localized in the cytosol and interacts with a G_i_ protein. Classically, CB1 is mainly located in central and peripheral nervous system and CB2 in immune cells, even though some neurons are able to express CB2 receptors [33].

AEA and 2-AG are synthesized “on demand” from lipophilic precursors and immediately released without being stored in vesicles [39]. Once bound to CB receptors, AEA activates a signal transduction pathway, resulting in blocking the production of the intracellular second messenger, cyclic adenosine 3′,5′-monophosphate (cAMP) [40,41]. Indeed, CB1 and CB2 receptors are G_i_ protein-coupled receptors that, once activated, are able to block most isoforms of the adenylate cyclase enzyme [42]. However, co-expression of CB1 or CB2 with adenylate cyclase isoforms 2, 4, or 7 resulted in stimulation of cyclic AMP accumulation [43], and may indicate a second method for cannabinoid activation to influence cellular processes.

Blocking of the adenylate cyclase enzyme inhibits the synthesis of cAMP; as result, the cellular activity is highly modulated. The main enzyme affected by lower levels of cAMP is the protein kinase A (PKA), a key cAMP-dependent enzyme involved in phosphorylation-mediated activation of several biochemical events inside the cell, including regulation of gene expression [44,45]. cAMP-dependent PKA is a heterotetramer composed of two regulatory (R) and two catalytic (C) subunits. The specificity and the versatility of the cAMP-PKA is due to the regulatory and the catalytic subunits that possess distinct physical/biological propertiesand are able to form different isoforms of PKA holoenzymes [44]. PKA is able to regulate several genes through a wide range of different transcription factors. Increased levels of cellular cAMP trigger the dissociation of the PKA heterotetramer, the C subunits migrate into the nucleus by passive diffusion and catalyze the phosphorylation of the cyclic AMP response element (CRE)-binding protein (CREB), allowing the transcription of genes controlling cellular metabolism (i.e., gluconeogenesis) and respiration [46]. cAMP-PKA enzyme is a key regulator of physiological processes such as activation of ion channels in the nervous system, regulation of the cell cycle (microtubule dynamics, chromatin condensation and decondensation, nuclear envelope dissambly and reassembly), and intracellular transport mechanisms [44]. Among the biological processes, cAMP-PKA signaling pathway is involved in diabetes insipidus and mellitus, hypertension, gastric ulcers, thyroid disease, asthma, in the control of metabolism in adipose tissue and in the regulation of steroidogenesis, reproductive function, and immune responses [44].

However, other enzymes regulated by CB1 activation include focal adhesion kinase, mitogen-activated protein kinase, phosphatidylinositol 3-kinase, and several enzymes involved in energy metabolism [48].

The enzyme *N*-acylphosphatidylethanolamine-hydrolyzing phospholipase D (NAPE-PLD) is a metallo-β-lactamase able to catalyze the hydrolysis of NAPEs, in this way forming AEA [49]. X-ray fluorescence analysis has revealed that the metal center of NAPE-PLD enzyme contains two zinc atoms. This binuclear metal center is responsible for binding and orienting the substrate for catalysis [50]. Once unbounded after being bind to the receptor, AEA is physiologically inactivated by uptake into the cells, followed by catalytic hydrolysis via fatty acid amide hydrolase (FAAH) [51]. NAPE-PLD and FAAH enzymes are the other components of the EC system.

## 4. EC System in Neuropsychiatric Disorders

The EC system plays a key role in several psychiatric disorders (i.e., anxiety, major depression, bipolar disorder and schizophrenia) [52]. Endocannabinoids, by modulating synaptic neurotransmission, are involved in the development of the central nervous system [53]. Indeed, 2-AG, through CB1 receptor activation and consequent ERK1/2 phosphorilation, is able to modulate synaptogenesis, axonal outgrowth, neuronal cell fate, migration and proliferation [53,54]. 2-AG shows a key role in post-traumatic stress disorder and memory [55]. It has been demonstrated that stimulating hippocampal CB1 receptors, directly through the synthetic cannabinoid receptor agonist WIN55212-2 or indirectly with a 2-AG hydrolysis inhibitor, is able to increase the spatial memory performance of rats trained under a higher stressful condition [55]. Interestingly, it has been proposed that the placenta, fetal adipose tissue and nervous tissues could interact via EC signals and that maternal nutrition during pregnancy could affect the formation and function of the hippocampus and hypothalamus by altering EC signaling [53]. It is likely that at the basis of this involvement in brain disorders there is the link between the EC system and neurotrophin signaling. Brain-derived neurotrophic factor (BDNF) and CB1 receptors cooperate to protect against excitotoxicity [56]. Genetic or pharmacological blockade of CB1 receptor increased neuron scusceptibility to kainic acid-excitotoxicity; interestingly, exogenous BDNF counteracted the damages of CB1 receptor inactivation, also preventing neuronal death [57]. Furthermore, CB1 receptor activation is able to induce the expression of immediate early genes, including *BDNF* mRNA [58]. A cooperation between CB1 and fibroblast growth factor (FGF) drives axonal growth [59]. CB1 receptor also shows neuroprotective capacities by decreasing tumor necrosis factor (TNF)-α levels in neurodegenerative conditions [60]. ECs are also linked to neurotransmitters, in that dopamine transmission and the EC system exhibit feedback controls on each other. Indeed, cannabinergic signaling is able to release dopamine, whilst dopaminergic signaling, via dopamine D2-like receptors, lead to up-regulation of EC signaling [61]. EC signaling also functions as a retrograde signaling system in GABAergic and glutamatergic synapses (inhibitory effect on glutamate) [62,63].

The fact that the EC system represents the link between immune and central nervous systems is also worth noting [64]. CB2 receptors are primarily located on immune system cells and serve as immune system modulators [65], while CB1 receptors are located in the central nervous system (particularly in cerebral cortex, hippocampus, basal ganglia, and cerebellum, lower levels are detectable in hypothalamus and spinal cord), peripheral nervous system, and peripheral organs [33]. ECs influence neuroimmune function and neuroinflammation and are also a key regulator of other metabolic and cellular pathways involved in autism, such as food intake, energy metabolism and control of the immune system.

## 5. ECs and Autism

In the CNS, CB1 receptors are expressed in the cerebellum, hippocampus, and the basal ganglia [66], which are areas in the brain implicated as dysfunctional in autism [67,68]. It has been demonstrated that during development, CB1 receptors drive axon guidance and are responsible for synaptogenesis [56,69,70]. Autistic children have been shown to have abnormal brain connectivity, which could be due to lack of CB1 axon guidance [71].

In the immune system, CB2 receptors act as modulators [64]. They are responsible for control of the movement of inflammatory cells to the site of injury [72]. CB2 receptor agonists are able to decrease TNF-α-induced human endothelial cell activation and transendothelial migration of monocytes by interfering with endothelial adhesion [73].

In the valproic acid (VPA) rat model of autism, CB1 receptors displayed altered phosphorylation in different brain areas associated with changes in AEA metabolism [74]. Interestingly, in VPA-exposed rats the expression of NAPE-PLD was reduced, whereas the expression of FAAH was increased, indicating a reduced AEA-mediated signaling that could be responsible for the deficits in the communicative and social domain. Furthermore, the administration of the AEA hydrolysis inhibitor URB597 ameliorated the social and behavioral deficits [74]. FAAH inhibition as strategy to increase social behaviors was further confirmed both in the VPA model and in an inflammatory rat model [75,76].VPA-exposed rats showed reduced peroxisome proliferator-activated receptor (PPAR)α/γ and orphan G protein-coupled receptor 55 (GPR55) expression in the frontal cortex and hippocampus [77]. These biomolecules are additional alternative receptor targets of the ECs involved in behavioral changes. It is to be considered that, like all animal models, VPA-exposed rodents do not fully replicate the human disease; however, this model provides a valuable tool to investigate the neurobiology underlying autistic behavior and to identify for novel therapeutic targets [78].

Several evidences demonstrate a key role for the EC system in ASD (Table 1). It was confirmed by Foldy and colleagues that found that neuroligin-3 mutations associated with autism commonly disrupt tonic EC signaling [79], as well as by in vitro data demonstrating that CB2 receptors are up-regulated (both mRNA and protein levels) in autistic-derived peripheral blood mononuclear cells [47]. Interestingly, the mRNA for the AEA-synthesizing enzyme NAPE-PLD was significantly decreased [47]. The simultaneous up-regulation of CB2 receptors and down-regulation of NAPE-PLD in these type of immune cells indicates that EC system drives immune-mediated changes in autism. More interesting, in vitro monocyte-derived macrophagic cells from individuals with ASD further display EC system dysregulation [80]. This indicates the involvement of the EC system in autism associated immunological disruptions, as macrophages are specialized cells strongly involved in inflammation responses [80]. Further, autistic in vitro monocyte-derived macrophages showed an increase in AEA-biosynthetic enzyme NAPE-PLD, together with a decrease in the AEA catabolic enzyme FAAH, indicating an overall increase in the EC AEA levels [80]. As natural agonist of CB2 receptors, AEA down-regulates cAMP production. Agonist-induced inhibition of adenylyl cyclase enzyme in cells expressing human CB2 receptors has been demonstrated [81].

Palmitoylethanolamide (PEA) is an endogenous *N*-acylethanolamine (the same molecular family of AEA), it has been shown to be an indirect cannabinoid agonist by decreasing the inactivation of the endocannabinoid anandamide [82]. Although PEA does not directly activate CB receptors, it has anti-inflammatory and anti-nociceptive properties [83]. The effects of FAAH enzyme inhibition in ameliorating autistic social behaviors could be also due to increase *N*-acylethanolamines such as PEA and oleoylethanolamine (OEA). Indeed, PEA and OEA are substrates degraded by FAAH enzyme [84]. It has been demonstrated that FAAH-deficient mice showed higher brain levels of AEA, PEA and OEA than those in wild-type mice [85]. As an anti-inflammatory molecule, PEA is able to reduce cyclooxygenase (COX) activity in macrophages in a model of inflammatory pain [83]. PEA has also effective intestinal anti-inflammatory characteristics [86], which is a point of interest for autism since part of the autistic chronic inflammatory state is mediated via the gastrointestinal associated immune system [10,87,88]. The observed anti-inflammatory effects of PEA are exerted through activation of CB2, GPR55 and PPAR receptors [86]. Very importantly, in the autism VPA mouse model, the administration of co-ultramicronized PEA in association with luteolin was effective on social and behavioral defects [89]. Treated VPA-induced autistic-like mice showed increased hippocampal neurogenesis and synaptic plasticity, as well as reduced expression of pro-inflammatory markers, and overall reduction in neuroinflammation. The same authors reported a case of an autistic child treated with PEA and luteolin; this treatment was able to reduce behavioral alterations [89]. It has been reported that ultramicronized PEA, administered alone, reduced inflammatory markers and produced rapid clinically significant improvements in two teenage boys with autism [90]. With the success of these case reports, appropriate double-blind controlled clinical trials to further explore the potential of cannabinoid agonists as a treatment for autism are encouraged.

Recently, a very interesting link between an analgesic drug, acetaminophen (*N*-acetyl-*para*-aminophenol), and social behaviors has been reported. Acetaminophen mechanism of action involves EC system. Indeed, its effects could be mediated by the active metabolite *p*-aminophenol, which in turn is conjugated with arachidonic acid by FAAH to form AM404. AM404 exerts effect through CB receptors. Local applications of acetaminophen promote social interactions in Swiss mice [91]. AM404 is structurally similar to AEA and shows weak agonist action on CB receptors, it also inhibits AEA-membrane transporter, in this way enhancing EC tone [92]. Conversely, it has been shown that the AM404 and *p*-aminophenol are toxic for mouse embryonic cortical neurons [93]. In addition, acetaminophen differentially changes social behavior in a mouse model of autism [94]. Neonatal exposure to acetaminophen affects cognitive function and alters its analgesic and anxiolytic response in adult male mice [95]. Prenatal and perinatal use of acetaminophen was linked to autism in an ecological study in 2013 [96] and an increased risk for autism from acetaminophen use in young children has been shown in a parental survey where parents reported confirmed diagnoses of ASD [97]. This increased risk for autism may be due to acetaminophen disruption of the EC system [98]. It is known that acetaminophen produces analgesia by an indirect agonist effect at cannabinoid receptors in the brain through conversion of the acetaminophen metabolite *p*-aminophenol to *N*-arachidonoylaminophenol [99,100,101]. Blocking cannabinoid receptors completely eliminates the analgesic effect of acetaminophen [102,103]. Furthermore, the events in the history of acetaminophen use have been related to autism and asthma [104]. The number of children with autism or asthma greatly increased in the US after the CDC issued a warning against using aspirin for children’s fever in 1980, which increased acetaminophen use. It has been shown that there were separate decreases in the number of children with autism or asthma born in the two years after two highly publicized US incidents in 1982 and 1986 where acetaminophen capsules were laced with cyanide [104] which reduced the use of acetaminophen. Asthma and autism both prominently feature an increased inflammatory state. Parker and colleagues have reviewed the association of acetaminophen and autism in a report published in 2017 [105]. Moreover, asthma and allergic inflammation conditions also show EC system involvement: CB2 could directly contribute to the pathogenesis of eosinophil-mediated diseases [106,107]. Taken together, all these findings highlight a controversial role for acetaminophen in ASD. No experimental studies demonstrate that prenatal acetaminophen exposure causes developmental brain alterations of progeny [108]. This paradoxical effect could be related to the doses of drug used: low doses could produce the neuroprotective effects [92].

Although ECs are attractive candidates for the restoration of ASD, several concerns must be addressed to adequately understand their proper application. The EC system plays a key role in the development of the central nervous system and its activation can induce long-lasting functional alterations [109]. Use of the exogenous cannabinoid tetrahydrocannabinol in the still-maturing brain may produce persistent alterations in brain structure and cognition [110]. Animal models have revealed long-lasting brain dysfunction and memory impairment as danger of both cannabis abuse and exposure to cannabinoid drugs during brain development [111]. In addition, disentangling the psychoactive and therapeutic effects of cannabioids could be an obstacle to the their therapeutic use. However, cannabidiol (CBD), the non-psychoactive phytocannabinoid, has shown several therapeutic activities (i.e., neuroprotection, immunomodulation, anti-oxidative and anti-inflammatory properties) [112,113] with no-side effects (including psychotropic activity [114].

## 6. Conclusions

Pharmacological approaches for autism are directed at symptoms, rather than the underlying pathogenesis. The EC system in autism orchestrates the apparent nexus of the peripheral and central neuro-immunologically mediated effects in autism. The newest studies suggest that pharmacological modulation of the EC system could represent a novel approach for autism treatment [115]. Among the potential EC targets, modulation of CB2 receptor signaling could offer a promising therapeutic option with minimal psychotropic effects [116]. FAAH inhibition could offer another pharmaceutical strategy, as well as PEA supplementation, since it is a natural compound produced in humans and could represent a novel future treatment.

EC system modulation has been shown to be an effective treatment in vivo and in vitro models, the adverse side effects of CB receptor agonism needs to be weighed against the clinical benefit to patients [117]. In addition, psychoactive chemical components of exogenous cannabinoids, i.e., Δ-9-tetrahydrocannabinol (Δ-THC), could impact the positive effects of the non-psychoactive components cannabidiol, cannabinol, and cannabigerol. While randomized controlled trials of cannabis-based medicine (CBM) have been performed for several pathologies, e.g., multiple sclerosis, demonstrating the effectiveness, safety and tolerance [114], randomized placebo-controlled double blind clinical trials in ASD are to be encouraged. 

The use of cannabis for medical purposes is associated with several short- and long-term neurological adverse effects [118]. Furthermore, as ASD comprises heterogeneous subtypes, all of which have a significant unmet clinical need, a more clearly defined standard of clinical endophenotype would be useful to address ASD heterogeneity and potential EC modulation [27]. Subgroups of ASD individuals with higher levels of inflammation may benefit more from the anti-inflammatory effects of drugs that increase cannabinoid levels, and this should be studied more closely in clinical trials. Before advocating the use of Δ-THC, CBD or other endocannabinoid-mimetic drugs for the treatment of autism, clinical trials need to be performed to establish whether there is a beneficial effect and to provide protocols for their therapeutic use including benefits depending on individuals’ cannabinoid receptor subtypes.

## Figures and Tables

**Table 1:** PubMed analysis of current literature limited to keywords “EC system disruption human ASD”.

First Author	Year	Title	Reference
Schultz	2008	Acetaminophen (paracetamol) use, measles-mumps-rubella vaccination, and autistic disorder: The results of a parent survey	[97]
Schultz	2010	Can Autism Be Triggered by Acetaminophen Activation of the Endocannabinoid System?	[98]
Becker	2010	Similarities in features of autism and asthma and a possible link to acetaminophen use.	[104]
Bauer	2013	Prenatal and perinatal analgesic exposure and autism: An ecological link	[96]
McFadden	2013	Evidence for dysregulation of axonal growth and guidance in the etiology of ASD.	[71]
Siniscalco	2013	Cannabinoid receptor type 2, but not type 1, is up-regulated in peripheral blood mononuclear cells of children affected by autistic disorders.	[47]
Siniscalco	2014	The in vitro GcMAF effects on endocannabinoid system transcriptionomics, receptor formation, and cell activity of autism-derived macrophages.	[80]
Parker	2017	The role of oxidative stress, inflammation and acetaminophen exposure from birth to early childhood in the induction of autism.	[105]
